# Guillain–Barré Syndrome Post COVID-19 Vaccination with ChAdOx1 nCoV-19 Vaccine: A Colombian Case Report

**DOI:** 10.1155/2023/3290956

**Published:** 2023-10-11

**Authors:** Isabel Cristina Hurtado, Raul Vallejo-Serna, Juan Sebastian Hurtado-Zapata, Sandra Patricia Misnaza

**Affiliations:** ^1^Grupo de Vigilancia en Salud Pública, Secretaría Departamental de Salud del Valle del Cauca, Cali, Colombia; ^2^Hospital Universitario del Valle Evaristo García, Cali, Colombia

## Abstract

**Background:**

Adverse events after vaccination against COVID-19 include rare events, such as Guillain–Barré syndrome. *Study Aims*. Documentation of clinical and temporary characteristics of the Guillain–Barré syndrome after using anti-COVID-19 ChAdOx1 nCoV-19 vaccine. *Case Presentation*. An adult, 29-year-old male, without relevant medical history, who developed neuromuscular symptoms nine days after administration of the first dose of anti-COVID-19 ChAdOx1 nCoV-19 vaccine.

**Results:**

Symptoms appeared nine days after vaccination, with lower limbs paresthesia. Three days later, paresthesia of upper limbs occurred. The following day, distal weakness of limbs, with standing and gripping difficulties, occurred. The clinical evaluation demonstrated dysarthria, incomplete palpebral closure, bilateral facial, and tongue paresis. The electromyography was compatible with a motor demyelinating polyneuropathy, confirming the diagnosis of the Guillain–Barré syndrome. Management with five sessions of plasma exchange was prescribed, with favorable clinical results.

**Conclusions:**

Clinical and laboratory tests confirmed the Guillain–Barré syndrome and the time elapsed from the date of the vaccine administration to the appearance of initial symptoms, added to the absence of other causes, and allowed to establish that the disease was caused by the vaccination.

## 1. Introduction

During the COVID-19 pandemic global emergency, diverse vaccines were authorized following manufacturing safety, efficacy, and quality standards demanded by the World Health Organization (WHO). As it is a new vaccine, rare adverse events are still being documented [1].

As anti-COVID-19 vaccination coverage increased with more timely and universal access, rare suspicious events were reported, such as Guillain–Barré syndrome (GBS) after vaccination, and more frequently, after nonreplicating viral vectors' vaccines. Therefore, the Pharmacovigilance Risk Assessment Committee of the European Medical Association recommended in its meeting of July, 2021, a change in the technical datasheet of AstraZeneca's vaccine against COVID-19 to include a warning so the health sector professionals and general public with access to that vaccine could be aware of the potential risk of acquiring the GBS [2], since some suspicious cases had been reported to the European Vigilance System, although they indicated that the available data did not confirm or discard a causal association [3].

Colombia made its first case report of this event after the application of the Pfizer-BioNTech vaccine in March, 2021 [4], and at global level, cases were reported after administration of the AstraZeneca vaccine in Brazil [5], Korea [6], India [7], Iran [8], Italy [9], the United Kingdom [10–14], and Tanzania [15], mostly in adults older than 50 years of age. Since the beginning of vaccine Pharmacovigilance in Colombia, no cases of GBS were reported after administration of the ChAdOx1 nCoV-19 vaccine in the national public health vigilance system [16].

Based on the abovementioned, the objective of this study was to document the Guillain–Barré syndrome case in a male, young adult, who started having symptoms nine days after receiving the initial dose of anti-COVID ChAdOx1 nCoV-19 vaccine.

## 2. Case Presentation

The case of a mestizo, 29-year-old male, who did not refer a medical history of interest or neurological family records before the vaccine administration, was reported. For this case report, the patient signed the informed consent, and the diagnosis was established according to Brighton collaboration criteria, based on the patient's clinical records.

## 3. Results

A mestizo, 29-year-old male, residing in the urban area of south-western Colombia (Tuluá, Valle del Cauca), without relevant medical records or infection history due to SARSCov2, except for childhood asthma, received the initial dose of ChAdOx1-S (Vaxzevria-AstraZeneca) on October 11, 2021. Nine days later, he showed clinical symptoms which initially included lower limbs paresthesia, followed by paresthesia of upper limbs after three days, accompanied by distal weakness in arms and legs, causing standing and gripping difficulties. On the 11th day after the vaccine, the patient had difficulty in remaining seated, associated to an occipital headache. Initially, the patient went to a local hospital, where vital signs were within normal ranges. Cardiopulmonary physical exam did not show any relevant findings, and there was no deterioration of his superior mental functions shown in the neurological assessment.

The patient had presence of dysarthria, incomplete palpebral closure due to eye orbicular muscle weakness, bilateral facial paresis with weakness of the buccinator and orbicularis muscle of the mouth, tongue with protrusion weakness on the left side, without affecting other cranial nerves, mainly proximal quadriparesis, osteotendinous reflexes +/++++, and bilateral neutral plantar response, without sensitive compromise. Studies were carried out, including blood count, kidney function, and normal electrolytes (sodium, potassium, calcium, magnesium, and phosphorus), HIV negative magnetic test, nuclear magnetic resonance (NMR) of the cervical and dorsal spine, normal skull CT-scan without contrast, and normal lumbar puncture opening pressure, with albumin-cytologic dissociation and normal glucose results (Table 1). For that reason, the patient was referred to a higher complexity institution, with suspicion of the Guillain–Barré syndrome. Electromyography compatibility with motor demyelination polyneuropathy was obtained. The neurology department's physicians evaluated the patient, who was considered to have an acute demyelinating polyneuropathy, Guillain–Barré syndrome-type. Prescription of management with five sessions of plasma exchange therapy was obtained, obtaining a favorable clinical response, leaving only a discrete weakness in the lower limbs, but being able to gait.

During the 18 month follow-up after the presentation of the adverse event, satisfactory evolution was evidenced, without diuresis or stool problems, without motor sequelae, with transitory paresthesia, and sensory sequelae in the first toe of the left foot. The patient did not receive additional doses of vaccine against COVID-19 or other biologics. Given the good evolution, it was not necessary to perform a control electromyography.

## 4. Discussion

Until May 2022, the epidemiological behavior of COVID-19 had a total of 531 million cases, with 6.2 million global deaths. In Colombia, according to official data from the National Health Institute, 6.1 million people were confirmed as infected with SARS-CoV-2 and 139,000 deaths, with a vaccination percentage of 70.31% [17, 18].

The Oxford/AstraZeneca ChAdOx1 nCoV-19 vaccine (later denominated Vaxzevria) was approved for its manufacturing and distribution by WHO during the COVID-19 pandemic. This vaccine uses a viral vector (a chimpanzee recombinant adenoviral vaccine) which expresses S antigen of the peak protein of SARS-CoV-2, inducing immunization against it. The most recent data from the Ministry of Health and Social Protection of Colombia indicate that as of May 2022, a total of 84,035,139 anti-COVID-19 vaccines had been administered, with 51,137 reports of serious and nonserious adverse events, for a rate of 61 postvaccine adverse events per 100,000 applied dosages. Out of the reported events, 8,019 corresponded to Vaxzveria secondary events, with a rate of serious adverse events estimated at 9.5 per 100,000 applied dosages [19].

Anti-COVID-19 vaccines have been related to a diversity of adverse effects, which include pain at the vaccine application site, general symptoms such as fatigue, edema, heart and liver disturbances, and neurological manifestations, such as seizures, impairment of consciousness, and GBS [14, 20]. Among the severe effects, polyneuropathies are the most reported conditions after the vaccine application. A systematic revision made in 2021 found within 17 evaluated articles, thirty-nine patients diagnosed with GBS, out of which 64.1% (25/39) correspond to events associated with the AstraZeneca vaccine, followed by BNT162b2 (Pfizer/BioNTech), with 31% (12/39) [21].

In the case of Vaxzevria vaccine, considering that antigen S can be united to the sialic acid constituting the glycoproteins and gangliosides present in the cell membranes, the crossed antibody reaction is presumed to be associated to the appearance of GBS in this vaccine [22, 23]. GBS is an immune entity that compromises peripheral nerves, being the most frequent cause for acute flaccid paralysis [24]. GBS usually occurs as a consequence of recent infections caused by *Campylobacter jejuni*, Zika virus, and *Mycoplasma pneumoniae*, among others, which generate an autoimmune disorder that attacks the spinal roots and peripheral nervous system [25]; this condition has also been described after administration of vaccines since 1970, when the first cases were documented, especially with vaccines against polio, hepatitis A and B, influenza, rabies, and currently, anti-COVID-19 vaccines [14]. In most cases, temporary associations between the initial symptoms and the vaccine administration were documented, without establishing their cause.

The classical presentation of GBS initiates with paresthesia and distal weakness in upper and lower limbs, with proximal progression, as in the case of our patient, with a clinical condition like those described by Maramattom et al. in India, where seven cases of GBS were reported after the administration of the ChAdOx1 nCoV-19 (Vaxzevria) vaccine, with facial paresis, quadriparesis, and areflexia [7]. Our patient also presented dysarthria, incomplete eyelid closing, and bilateral facial and tongue paresis. In our case, symptoms started nine days after vaccination, relating to findings in international publications of anti-COVID-19 vaccines, which mention periods between seven and 22 days, including Vaxzevria [21]. When the GBS was diagnosed, our patient was 29 years old, an age lower than that observed in other cases reported in Europe, the United States, and Asia, where the average patient's age was between 47 and 69 years of age [14, 21, 26]. The literature reports that the initial diagnose of SGB is clinical, and later it is supported by findings in the lumbar puncture (spinal tap) and electrodiagnostic testing, where albumin-cytologic dissociation and abnormalities are consistent with GBS [27] in the same way that it was presented in our case.

Until this date in Colombia, only two cases of GBS have been documented after vaccination against COVID-19, both of which were secondary to CoronaVac® [28, 29]. In these cases, GBS presentation was the classic one, with initial quadriparesis. One of the patients had ventilatory failure, requiring orotracheal intubation, which was associated to the age (73 years old); this patient was older than those observed in other publications. These cases presented between nine and 21 days after the vaccine application, coinciding with our case report.

In conclusion, the clinical data and lab exams confirmed the GBS diagnosis and the time between administration of the vaccine and appearance of symptoms, as well as the absence of other causes, allowing the classification of this case as an adverse event following immunization (AEFI).

## Figures and Tables

**Table 1:** Description of patient lab exams, October to November, 2021.

Exam	Result
Albumin	4.2 g/dl

Neuroaxis simple and contrast NMR	Normal

Blood count	Leukocytes 13,900; N 10,430; L 2,230; Hb 16 g/dl; hematocrit 49%; platelets 367,000

Ionogram	K: 4.4 mmol/L, Na: 141 mmol/L, Ca: 9.8 mg/dl, Mg: 2.51 mg/dl, P: 5.79 mg/dl

Kidney function	Creatinine 0.66 mg/dl, blood urea nitrogen 19.4 mg/dl

Electromyography	Absent bilateral peroneal and tibial nerve conduction blockage. Sensitive nerve conduction of the medial ulnar, and the sural bilateral nerve with normal conduction latencies, amplitudes and speeds, prolonged latency of bilateral medial and ulnar, normal amplitudes, absent *f* wave in bilateral peroneal nerve, electromyography of muscles, with insertional activity and normal recruitment, almost complete interference pattern, normal motor units, and without signs of membrane instability. Electrophysiological evidence of a motor demyelinating polyneuropathy

Findings in cerebrospinal fluid	Color: colorless
	Polymorphonuclears: 2/mm^3^
	Lymphocytes: 2/mm^3^
	Erythrocytes: 0/mm^3^
	Proteins: 103 mg/dl
	Glucose: 53 mg/dl

COVID-19 antigen	Negative

## Data Availability

The data used to support the findings of this study are available from the corresponding author on request.
